# Two-Dimensional Cr_5_Te_8_@Graphite Heterostructure for Efficient Electromagnetic Microwave Absorption

**DOI:** 10.1007/s40820-023-01271-7

**Published:** 2023-12-20

**Authors:** Liyuan Qin, Ziyang Guo, Shuai Zhao, Denan Kong, Wei Jiang, Ruibin Liu, Xijuan Lv, Jiadong Zhou, Qinghai Shu

**Affiliations:** 1 School of Materials Science and Engineering, Beijing Institute of Technology, Beijing, 100081 People’s Republic of China; 2 Centre for Quantum Physics, Key Laboratory of Advanced Optoelectronic Quantum Architecture and Measurement (MOE), School of Physics, Beijing Institute of Technology, Beijing, 100081 People’s Republic of China; 3 Advanced Research Institute of Multidisciplinary Science, Beijing Institute of Technology, Beijing, 100081 People’s Republic of China; 4 Tangshan Research Institute, Beijing Institute of Technology, Tangshan, 063099 People’s Republic of China

**Keywords:** Chemical vapor deposition, Interface polarization engineering, Cr_5_Te_8_-graphite heterojunctions, Microwave absorption

## Abstract

Two-dimensional (2D) transition metal chalcogenides (TMCs) hold great promise as novel microwave absorption materials owing to their interlayer interactions and unique magnetoelectric properties. However, overcoming the impedance mismatch at the low loading is still a challenge for TMCs due to the restricted loss pathways caused by their high-density characteristic. Here, an interface engineering based on the heterostructure of 2D Cr_5_Te_8_ and graphite is in situ constructed via a one-step chemical vapor deposit to modulate impedance matching and introduce multiple attenuation mechanisms. Intriguingly, the Cr_5_Te_8_@EG (ECT) heterostructure exhibits a minimum reflection loss of up to − 57.6 dB at 15.4 GHz with a thin thickness of only 1.4 mm under a low filling rate of 10%. The density functional theory calculations confirm that the splendid performance of ECT heterostructure primarily derives from charge redistribution at the abundant intimate interfaces, thereby reinforcing interfacial polarization loss. Furthermore, the ECT coating displays a remarkable radar cross section reduction of 31.9 dB m^2^, demonstrating a great radar microwave scattering ability. This work sheds light on the interfacial coupled stimulus response mechanism of TMC-based heterogeneous structures and provides a feasible strategy to manipulate high-quality TMCs for excellent microwave absorbers.

**Supplementary Information:**

The online version contains supplementary material available at 10.1007/s40820-023-01271-7.

## Highlights

- A Cr_5_Te_8_@expanded graphite heterostructure is fabricated by chemical vapor deposition, exhibiting remarkable microwave absorption performance with a minimum reflection loss of up to − 57.6 dB at a thin thickness of only 1.4 mm under a low filling rate of 10%.
- Density functional theory calculations deeply reveal the polarization loss mechanism triggered by heterogeneous interfaces.
- The heterostructure coating displays a remarkable radar cross section reduction of 31.9 dB m^2^, demonstrating a great electromagnetic microwave scattering ability and radar stealth capability.

**Supplementary Information:**

The online version contains supplementary material available at 10.1007/s40820-023-01271-7.

## Graphical abstract

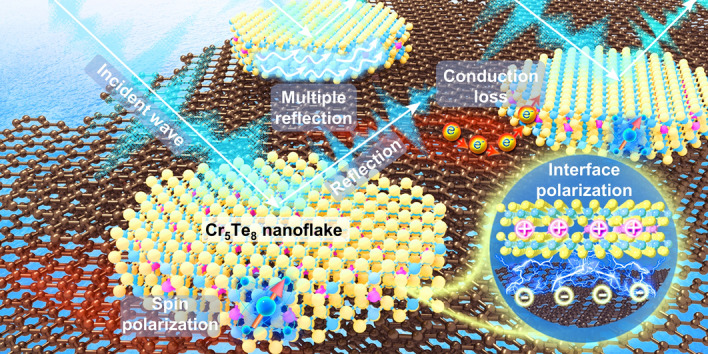

## Introduction

Electromagnetic microwave absorption (EMA) materials hold importance in the prevention of electromagnetic contamination produced by electronic productions [1–3]. Recently, 2D materials such as graphene, MXenes, and transition metal chalcogenides (TMCs) with exceptional properties have emerged as promising EMA materials, shining brightly in electromagnetic protection [4–7]. Unfortunately, pure materials exhibit poor EMA performance at a low loading due to the impedance mismatch caused by the deficiencies of interfacial polarization, dipole polarization and conductive loss [8–11].

Manipulating simultaneously chemical components and structural engineering is highly effective in achieving suitable electromagnetic parameters, leading to positive impedance matching and collaborative loss for the fabrication of advanced absorbers [12, 13]. TMCs offer a unique platform to explore novel electromagnetic protection properties benefiting from their layered structure, strong spin–orbit coupling [14], and various electronic band structures [15]. It is worth noting that the synthetic methods for high-quality TMCs are mainly based on high-temperature solid-state reactions (such as chemical vapor deposit (CVD) [16, 17] and solid-phase sintering and recrystallization [18, 19]). Indeed, high-quality TMCs crystals can bring excellent intrinsic magnetoelectric properties for an outstanding candidate of microwave attenuation components [12, 20]. For instance, Huang et al*.* [18] synthesized the high-quality ferromagnet Fe_3_GeTe_2_ absorber with a minimum reflection loss (RL) value of − 34.7 dB with a thickness of 5.5 mm at the loading of 70 wt%. Among other TMCs, chromium telluride (Cr_*m*_Te_*n*_) is one of the most representative Chromium-based chalcogenides (Cr_*m*_X_*n*_, where *X* = S, Se, and Te) owing to its tunable energy band structure via stoichiometric variations and magnetic properties derived from the strong spin–orbit coupling of Te atoms [16], which demonstrates that pushing forward Cr_*m*_Te_*n*_ into the novel absorber research is highly feasible and holds significant promise. However, the inherent characteristic of high density makes it challenging to easily manipulate TMCs into excellent absorbers, as it hinders overcoming the impedance mismatch due to the restricted attenuation channels at the low loading [20]. Therefore, the structural engineering design is a feasible strategy for TMCs to broaden the territory of electromagnetic attenuation abilities which is important to design the next generation of EMA materials.

Impressively, the prominent merits of heterointerface engineering inject infinite vitality into the design of high-efficiency and stimuli-responsive microwave absorbers [21, 22]. The TMCs-based heterostructures not only inherit the distinctive electromagnetic characteristics of intrinsic components but also introduce a range of compelling physicochemical properties. These properties fundamentally affect the polarization loss, conduction loss, and magnetic response due to the charge rearrangement and electron transport [21, 23]. Consequently, selecting the materials with suitable band structures to construct heterojunctions provides an opportunity for realizing the high-performance microwave absorbers at a low loading [21, 24]. However, previous reports on TMC-based heterostructures mainly focus on optimizing magnetoelectric components to enhance the EMA performance but in-depth and systematic studies are often lacking [12, 19, 25]. Thereby, designing a novel heterostructure and deeply excavating stimulus response polarization mechanism are urgent and significant for achieving the high-quality TMC-based EMA materials with excellent properties.

Herein, we ingeniously utilize the intrinsic property advantages of Cr_5_Te_8_ to establish a “face-to-face” heterostructure between 2D Cr_5_Te_8_ and graphite (EG) via a simple and efficient one-step CVD. Intriguingly, the optimal Cr_5_Te_8_@EG (ECT) shows superior EMA performance which the minimum RL can reach − 57.6 dB at 15.4 GHz with a thin thickness of 1.4 mm under a low filling rate of 10%. Density functional theory (DFT) calculations confirm the abundant interface coupling polarization between Cr_5_Te_8_ and EG owing to charge redistribution and energy band alignment. Besides, radar cross section (RCS) simulations prove that ECTs possess excellent electromagnetic loss capability under different plane microwave angles in near-practical situations, indicating the potential applications of ECTs in electronic communication devices. This work provides an efficient strategy for fabricating 2D TMC-based EMA materials and offers a valuable reference for in-depth microscopic analysis of the polarization loss mechanism.

## Experimental Section

### Materials

Te powders (99.999%), CrCl_3_ powders (99.9%), and primary expandable graphite (PEG) powders were purchased from Beijing InnoChem Science & Technology Co., Ltd. (China).

### CVD Synthesis of 2D Cr_5_Te_8_

CrCl_3_ and Te powders were used as precursors, and Si/SiO_2_ wafer was used as the substrate. A quartz boat loaded with Te powders was placed in upstream of the hot zone, where the temperature was about 500 °C. Subsequently, a ceramic boat containing CrCl_3_ powders was covered by a Si/SiO_2_ substrate. The boat was then placed at the heating center of the one-inch-diameter quartz tube. Before the heating process, high-purity argon (Ar, 500 sccm) gas was loaded to purge the reaction chamber for 5 min. After that, a mixture of Ar and H_2_ (100/5 sccm) was used as the carrier gas. The growth temperature was set to 730 °C with a ramp rate of 50 °C min^−1^ and maintained for 15 min for the growth of Cr_5_Te_8_. Then, natural cooling was adopted when the reaction was completed.

### CVD Preparation of ECT-1

40 mg CrCl_3_ powders and 10 mg PEG powders were well-mixed and thinly spread in a quartz boat, which PEG powers serve as the role of substrates. Then, this quartz boat was placed at the heating center of the one-inch-diameter quartz tube. A quartz boat loaded with Te powders was placed in upstream of the hot zone, where the temperature was about 500 °C. Before the heating process, high-purity argon (Ar, 500 sccm) gas was loaded to purge the reaction chamber for 5 min. After that, a mixture of Ar and H_2_ (100/5 sccm) was used as the carrier gas. The growth temperature was set to 730 °C with a ramp rate of 50 °C min^−1^ and maintained for 15 min for the growth of ECT-1. Then, natural cooling was adopted when the reaction was completed. ECT-2 and ECT-3 with different mass ratios of CrCl_3_ powders to PEG powers (1:1 and 1:4, respectively) were prepared by the same method.

### Preparation of EG

PEG powders were put into a quartz boat and then placed at the heating center of the one-inch-diameter quartz tube. Before the heating process, high-purity argon (Ar, 500 sccm) gas was used to purge the reaction chamber for 5 min. Then, the furnace temperature was ramped to the expandable temperature of 730 °C, with a mixture gas of 100 sccm Ar and 5 sccm H_2_, and maintained for 15 min for the growth of EG.

### Characterizations

The morphology and thickness of CVD-grown Cr_5_Te_8_ were characterized by OM (BX51, Olympus) and atomic force microscopy (AFM, Bruker ICON microscope). Scanning electron microscopy (SEM) was obtained by using Zeiss Ultra-55. Transmission electron microscopy (TEM) and high-resolution TEM (HRTEM) images were obtained by using JEOL JEM2100F operated at the accelerating voltage of 200 kV. Raman spectra was obtained on a Witec Raman spectroscope, with a 532 nm laser excitation. X-ray diffraction (XRD) patterns were obtained by using an x-ray diffractometer (Bruker D8 Advance) operated at 40 kV and 40 mA using Cu-Kα as the irradiation source (*λ* = 1.54060 Å). All samples were scanned at 0.8 s/step with a step size of 0.02° within the range of 5–90°. X-ray photoelectron spectroscopy (XPS) was conducted by using scanning x-ray microprobe (Thermo Scientific K-Alpha +) using Al K*α* radiation and the C1s peak at 284.8 eV as internal standard. Nitrogen adsorption–desorption isotherms were measured by an accelerated surface area and pore size analyzer (ASAP, Micromeritics TriStar II 3020). The pore size distributions were measured by the Barrett–Joyner–Halenda (BJH) method. Electromagnetic parameters were measured by an Agilent PNAN5244A vector network analyzer in the 2–18 GHz range scope. The measured materials were prepared by homogeneously mixing the absorbents with the paraffin matrix by the mass fraction of 10% and compacted into a coaxial ring of 7.00 mm outer diameter and 3.04 mm inner diameter. According to the transmission line theory, EMA intensity is defined by complex permittivity (*ε*_r_ = *ε*′ − *jε*″) and complex permeability (*µ*_r_ = *µ*′ − *jµ*″) [26, 27]. The RL values can be calculated by the following equations [28, 29]:

1 $$Z_{{{\text{in}}}} = Z_{0} \sqrt {\frac{{\mu_{r} }}{{\varepsilon_{r} }}} \tanh \left( {\frac{2\pi fd}{c}\sqrt {\frac{{\mu_{r} }}{{\varepsilon_{r} }}} } \right)$$

2 $${\text{RL}} = 20\log |\frac{{Z_{{{\text{in}}}} - Z_{0} }}{{Z_{{{\text{in}}}} + Z_{0} }}|$$

where *ε*_*r*_ and *μ*_*r*_ represent the complex permittivity and complex permeability, respectively, *d* is the measured sample thickness, *Z*_in_, *Z*_0_, *c*, and *f* are the input impedance, free space impedance, speed of light, and measured frequency, respectively.

Impedance matching (*Z*) is used to describe the ability of electromagnetic microwaves to enter the absorbents, which can be accessed as follows [23]:

3 $$Z = \frac{{Z_{{{\text{in}}}} }}{{Z_{0} }} = \sqrt {\frac{{\mu_{r} }}{{\varepsilon_{r} }}} \tanh \left( {\frac{2\pi fd}{c}\sqrt {\frac{{\mu_{r} }}{{\varepsilon_{r} }}} } \right)$$

### RCS Simulation

CST Studio Suite 2021 was used for simulating the radar cross-sectional of the magnetic-dielectric ECT. According to the metal back model, the simulation model of the specimens was established as a square (10 × 10 cm^2^) with dual layers, which consisted of an ECT absorption layer and a back plate of the perfect electric conductor (PEC). The thickness of the bottom PEC layer was 1.0 mm, and the absorber layer thickness values were set as 1.4 mm at the frequency of 15.4 GHz, respectively. The ECT-PEC model plate is placed on the X-O-Y plane and linear polarized plane electromagnetic microwaves incident from the positive direction of the *Z*-axis to the negative direction of the *Z*-axis. Meanwhile, the direction of electric polarization propagation is along the *X*-axis. Open boundary conditions are set in all directions with a field monitor frequency of 15.4 GHz. The RCS values can be described as follows [7]:

4 $$\sigma \left( {{\text{dBm}}^{2} } \right) = 10\log \left( {(4\pi S/\lambda^{2} )|E_{s} /E_{i} |} \right)^{2}$$

Here, *S* is the area of the target object simulation model, *λ* is the wavelength of the electromagnetic microwave, and *E*_s_ and *E*_i_ represent the electric field intensity of the scattered microwave and the incident microwave, respectively.

### Computational Methods

The static theoretical calculations were performed using spin polarized DFT as implemented in the Vienna ab initio simulation package [31]. The projected augmented wave (PAW) method was applied to treat the electron–ion interactions. The Perdew–Burke–Ernzerhof (PBE) exchange correlation functional within a generalized gradient approximation (GGA) was employed [32, 33]. Dispersion correction was applied in all calculations with the zero-damping DFT + D3 method of Grimme [34]. The valence electrons were expanded in a plane wave-cutoff basis set with a cutoff energy of 400 eV. The energy and force convergence were set to 10^–5^ eV and 0.02 eV Å^−1^ during the structure relaxation. The Brillouin zone was sampled using 3 × 3 × 1 Monkhorst–Pack *k*-point mesh in the geometrical optimization, while a 6 × 6 × 1 grid was used in the electronic structure calculations. Bader charge analysis was performed using the code developed by Henkelman’s group [35]. Coulomb interaction energy for Cr *d* orbitals was self-consistently determined to *U*_eff_ = 4.94 eV applied to Dudarev’s approach [36].

## Results and Discussion

### Synthetic Strategy and Structural Characterization

**Fig. 1:**
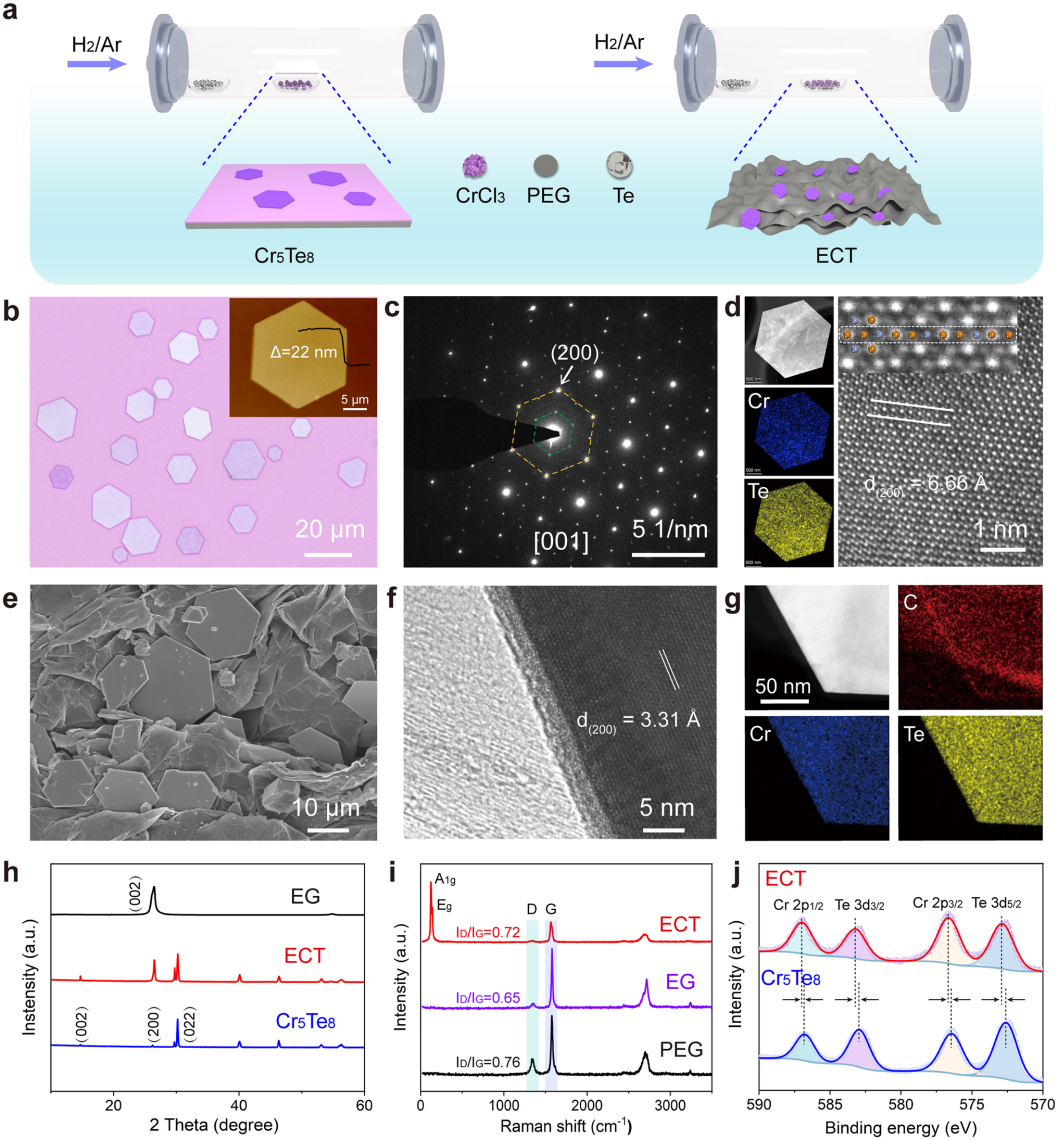
Synthetic strategy and structure characterization of 2D Cr_5_Te_8_ and ECT. **a** Schematic representation of the CVD method for the growth of Cr_5_Te_8_ and ECT. **b** Optical and AFM image of Cr_5_Te_8_ nanoflakes. **c** Diffraction patterns of Cr_5_Te_8_ crystals. **d** EDS mapping and HRTEM image of Cr_5_Te_8_ crystals (orange represents Te atoms, blue represents Cr atoms), showing the high-quality Cr_5_Te_8_ crystals were successfully synthesized by CVD. **e–g** SEM, HRTEM, and TEM-EDS mapping images, respectively. **h–j** XRD patterns, Raman spectra, and XPS spectra, respectively, demonstrating the successful preparation of ECT van der Waals heterojunction. (Color figure online)

To precisely and controllably synthesize Cr_5_Te_8_ crystals, an efficient CVD was applied, and the experimental setup is schematically shown in Fig. 1a. Briefly, tellurium (Te) and chromium trichloride (CrCl_3_) powders were used as precursors to directly synthesize 2D Cr_5_Te_8_ nanoflakes and ECT.

Figure 1b displays the optical image of the synthesized 2D Cr_5_Te_8_ crystals. AFM analysis indicates a flat surface with an approximate height of 22 nm. Moreover, two characteristic peaks at 124 and 141 cm^−1^ correspond to the out-of-plane A_1g_ and in-plane E_g_ of Cr_5_Te_8_, respectively, which is consistent with the previously reported results (Fig. S1a) [16]. XRD pattern in Fig. S1b shows that the diffraction peaks are in good agreement with the standard card of PDF#50-1153, suggesting that Cr_5_Te_8_ belongs to the p-3m1 space group with a trigonal crystal structure. Subsequently, the composition and atomic structure of Cr_5_Te_8_ were further examined by selected area diffraction and HRTEM. The crystal structure of trigonal Cr_5_Te_8_ can be regarded as Cr atoms intercalated in the van der Waals interlayer of CrTe_2_ [16]. The intercalation of Cr atoms leads to a new periodicity with the same orientation but half the frequency of the CrTe_2_ lattice in reciprocal space. This effect can be distinctly observed in the diffraction pattern from the [001] zone axis of Cr_5_Te_8_ [17]. As shown in Fig. 1c, the (200) spot is derived from the CrTe_2_ backbones and the inner (100) supercell spot comes from the intercalated Cr atoms. Meanwhile, the lattice spacing marked by the white line in the HRTEM image is 6.66 Å, which is twice the interplanar spacing of the (200) plane (Fig. 1d). Additionally, the atomic model shown in the magnified view of the HRTEM image reveals an alternating arrangement of Te and Cr atoms, which is confirmed by the intensity line profile extracted from the rectangular region marked with dashed lines (Fig. S1c). Furthermore, the HAADF image of Cr_5_Te_8_ and the corresponding electron diffraction spectrum (EDS) elemental mapping manifest the homogeneous spatial distribution of Cr and Te. The above characterizations demonstrate the high quality of as-synthesized Cr_5_Te_8_ crystals.

Subsequently, structural analysis was performed on the Cr_5_Te_8_@EG heterostructure. Figure 1e displays that Cr_5_Te_8_ nanoflakes are randomly anchored on EG. As shown in Fig. 1f, the lattice fringe of 3.31 Å corresponds to the (200) facet of trigonal Cr_5_Te_8_. The associated EDS elemental mapping illustrates the homogeneous distribution of Cr and Te elements in the Cr_5_Te_8_ crystals, while the C element identifies interfacial coupling (Fig. 1g). Significantly, the obvious lattice mismatch and distortion in the heterointerface region of the Cr_5_Te_8_ and EG phases will be beneficial in increasing the interfacial polarization relaxation. Moreover, the XRD patterns in Fig. 1h unveil distinct (002) and (004) characteristic peaks of Cr_5_Te_8_ and EG, respectively, indicating their well-defined layered structure. Notably, the XRD and Raman peaks of Cr_5_Te_8_ and EG were both identified in the ECT, implying the successful construction of a van der Waals heterojunction. Importantly, the value of *I*_D_/*I*_G_ for ECT is larger than that of EG (0.72 vs 0.65), suggesting that more defects are produced (Fig. 1i) [37]. Then, XPS pattern was further used to characterize the heterostructure (Fig. 1j). Two peaks located at a binding energy of 583.27 and 572.91 eV can be assigned to Te 3*d*_5/2_ and Te 3*d*_3/2_ [38], respectively, while the peaks at 587.02 and 576.67 eV can be attributed to Te 3*d*_5/2_ and Te 3*d*_3/2_ [39]. Intriguingly, compared with pure Cr_5_Te_8_, both peaks of Cr and Te in ECT shift to higher binding energy, affirming electron transfer from Cr_5_Te_8_ to the EG. In short, these results elaborate on the successful construction of ECT van der Waals heterojunction and the directed flow of electrons through the intimate interface, leading to changes in the electronic structure and redistribution of spatial charges.

### High-Efficiency EMA Properties

The frequency dependence of the RL values of Cr_5_Te_8_ and EG was investigated with different filler loadings, as shown in Fig. S2. It can be clearly seen that both Cr_5_Te_8_ and EG exhibit poor EMA performances for the following reasons: (i) Inappropriate electromagnetic parameters lead to unmatched impedance [40, 41]. According to Eq. (3) in the Experimental Section, a better impedance match is achieved when the values of |*Z*_in_/*Z*_0_ − 1| are closer to zero [42]. Unfortunately, as shown in Fig. S3a, b, the |*Z*_in_/*Z*_0_ − 1| values of Cr_5_Te_8_ and EG are far from zero. (ii) For pure Cr_5_Te_8_ or EG, the loss mechanism is relatively single, resulting in poor EMA ability.

**Fig. 2:**
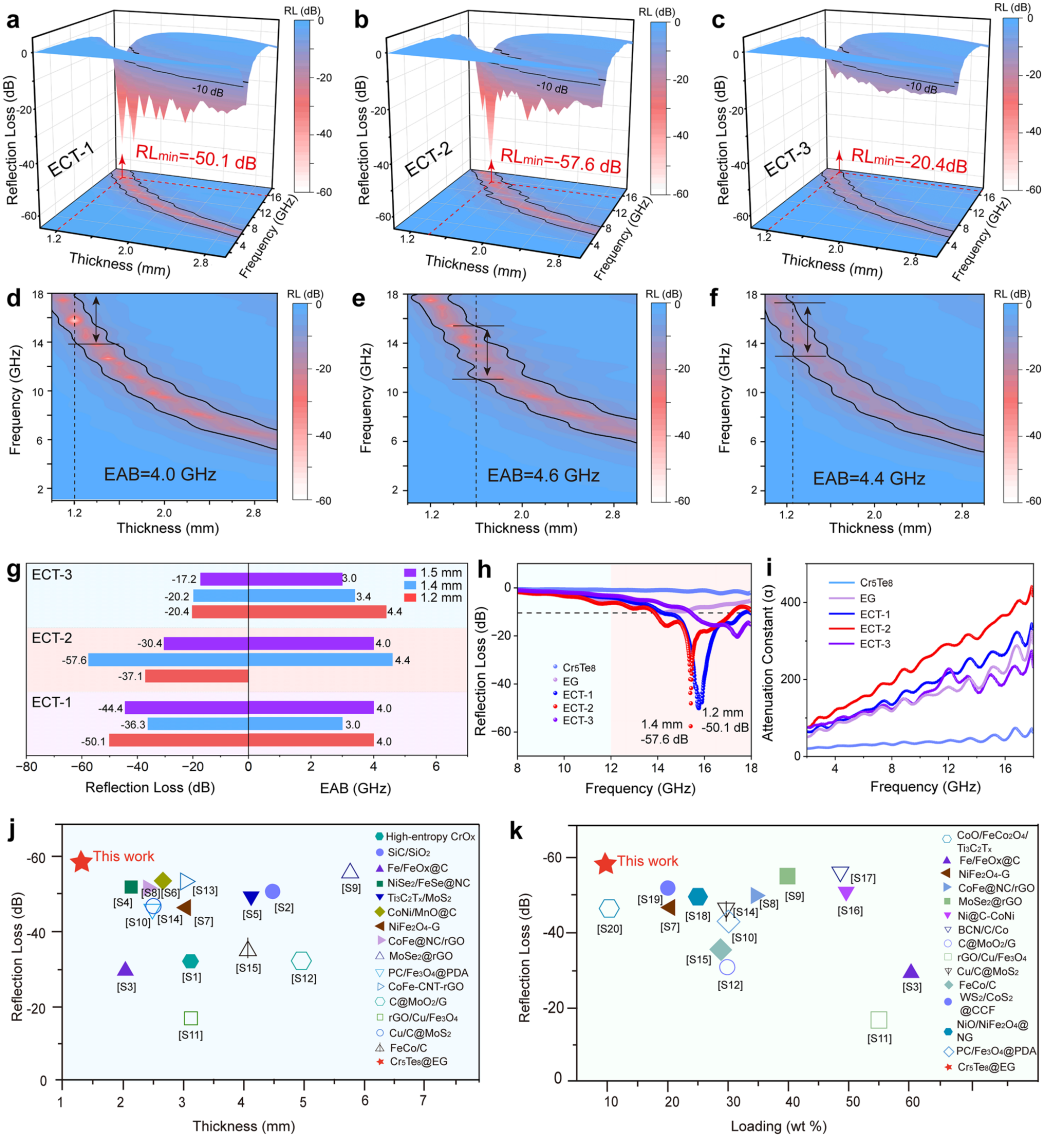
EMA performances of the Cr_5_Te_8_, ECT-1, ECT-2, ECT-3, and EG. **a, d** 3D RL values and corresponding contour maps of ECT-1, respectively. **b, e** 3D RL values and corresponding contour maps of ECT-2, respectively. **c, f** 3D RL values, and corresponding contour maps of ECT-3, respectively. All ECTs show excellent EMA performance. **g** RL and EAB values of ECT-1, ECT-2, and ECT-3. **h** RL values comparison of CT-1.4mm, ECT-1-1.2mm, ECT-2-1.4mm, ECT-3-1.2mm, and EG-1.4mm, indicating that ECTs with the heterostructure have superior EMA performance than pure EG or Cr_5_Te_8_. **i** The attenuation constant *α* curves. **j** Comparison of the minimum RL and corresponding thickness among different absorbers. See Refs. [S1-S15] in Table S1. **k** Comparison of the minimum RL and corresponding loading among different absorbers (see the references in Table S2), indicating that ECT has better absorption performance than other absorbers under low loading rate and thin thickness (wt% represents the loading rate of the absorber mixed with paraffin). (Color figure online)

**Fig. 3:**
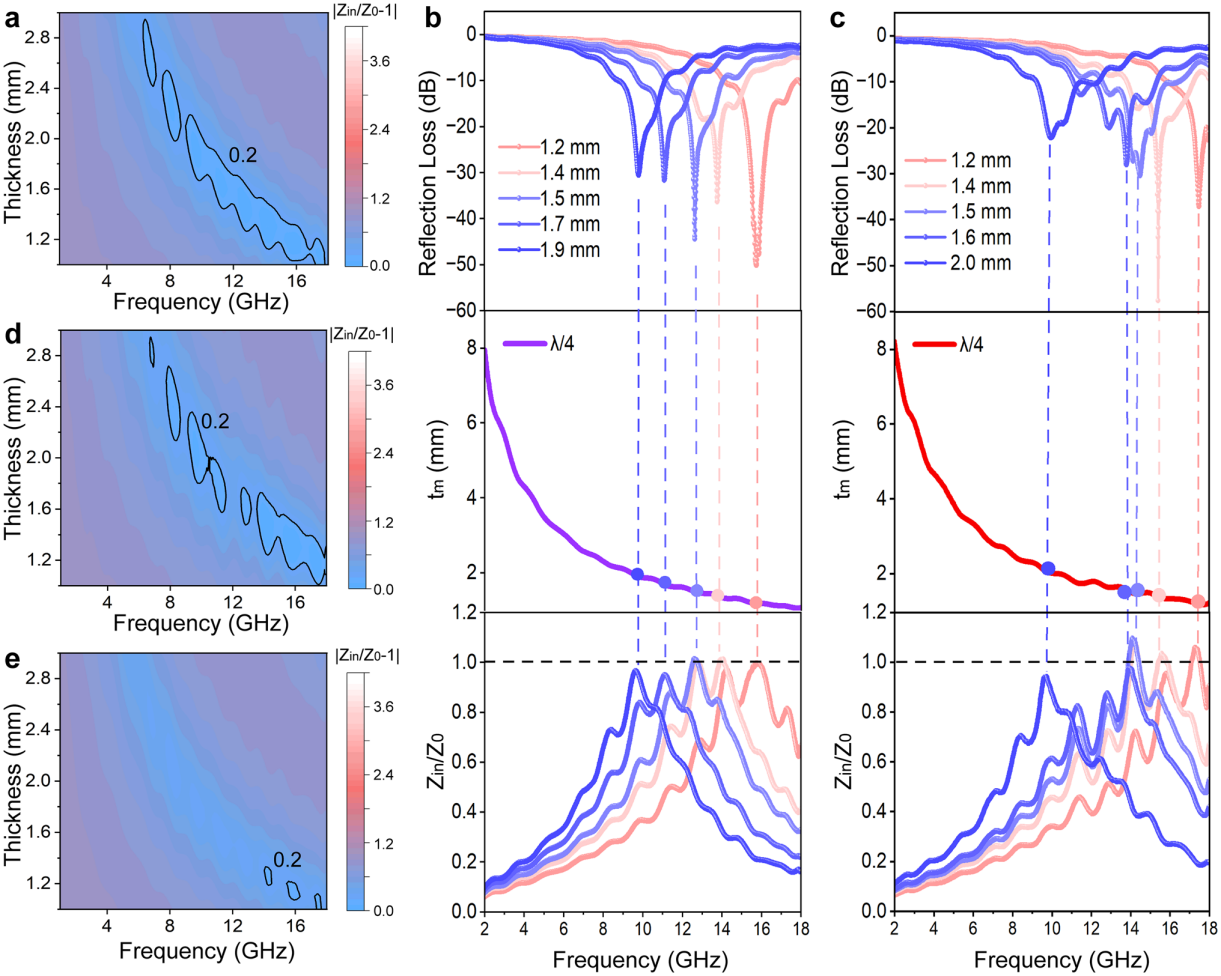
Impedance matching capability of ECTs. Impedance matching curves under different thicknesses of ECT-1 (**a**), ECT-2 (**d**), and ECT-3 (**e**), respectively. The difference between the *Z* values of ECT and the value of 1 is less than 0.2, showing the good impedance matching ability of ECTs. **b, c** RL-f, *t*_m_ − *f*, and |*Z*_in_/*Z*_0_|-f curves of ECT-1 and ECT-2 at different thicknesses (the filled ellipse represents the experimental matching thickness value), respectively, indicating ECT-1 and ECT-2 match well with the *λ*/4 matching model. (Color figure online)

Notably, the construction of Van der Waals heterojunctions can indeed enhance the final EMA performance (Fig. 2) and balance the impedance matching (Fig. 3). Compared with pure Cr_5_Te_8_ and EG, the minimum RL values of ECT-1, ECT-2, and ECT-3 significantly increase to − 50.1 dB at 1.2 mm, − 57.6 dB at 1.4 mm, and − 20.4 dB at 1.2 mm, respectively (Fig. 2a-c). Besides, the effective absorption bandwidth (EAB) values of ECTs are 4.0, 4.6, and 4.4 GHz, respectively (Fig. 2d-f). Figure 2g-h provides a more intuitive comparison of EMA performances, showing that ECTs have superior RL values and EAB at different thicknesses. Intriguingly, Fig. S4 shows that the EMA performance of ECTs far exceeds that of the physical mixture of EG and Cr_5_Te_8_, manifesting that the heterojunction plays a positive role in improving the absorption performance. Moreover, the attenuation constant α is another key metric for evaluating EMA performance. It can be defined as [43]:

5 $$\alpha = \left( {\sqrt 2 \pi f/c} \right)\sqrt {\mu^{\prime\prime}\varepsilon^{\prime\prime} - \mu^{\prime}\varepsilon^{\prime} + \sqrt {\left( {\mu^{\prime\prime}\varepsilon^{\prime\prime} - \mu^{\prime}\varepsilon^{\prime}} \right)^{2} + \left( {\mu^{\prime}\varepsilon^{\prime\prime} + \mu^{\prime\prime}\varepsilon^{\prime}} \right)^{2} } }$$

As shown in Fig. 2i, the successive decrease in* α* values of ECT-2, ECT-1, ECT-3, EG, and Cr_5_Te_8_ indicates a decrease in attenuation ability, which is consistent with the trend of their RL curves. In addition, the comparison of the minimum RL with different thicknesses (Fig. 2j) and different filler loadings (Fig. 2k) among various EMA materials illustrate that our ECT has an excellent EMA ability at a thinner thickness and a lower filler loading.

Evidently, correspondingly to the good EMA performances of ECTs is their great impedance matching degree. As shown in Fig. 3a, d, e, the |*Z*_in_/*Z*_0_ − 1| values of ECTs are close to the value of zero, indicating the input impedance of ECTs absorbers matches well with the free space impedance. Specifically, according to the quarter wavelength (*λ*/4) matching model, the simulated thickness (*t*_m_) values of absorbers are estimated by the equation: *t*_*m*_ = *nc*/$\left( {4 fm\sqrt {|\mu_{r} ||\varepsilon_{r} |} } \right)$ (*n* = 1, 3, 5,…) [25, 44]. Figure 3b, c displays that the minimum RL values are obtained at a frequency where |*Z*_in_/*Z*_0_| is much closer to the value of 1, and the experimental matching thicknesses values exactly fall on the *λ*/4 curve, indicating that electromagnetic microwaves can be easily entered into the ECT absorber.

### Multivariate Synergistic Loss Mechanism

Heterointerface structures and magnetic/dielectric components endow ECTs with stimulative impedance matching and efficient EMA. The detailed mechanisms can be clarified by the following four aspects.

**Fig. 4:**
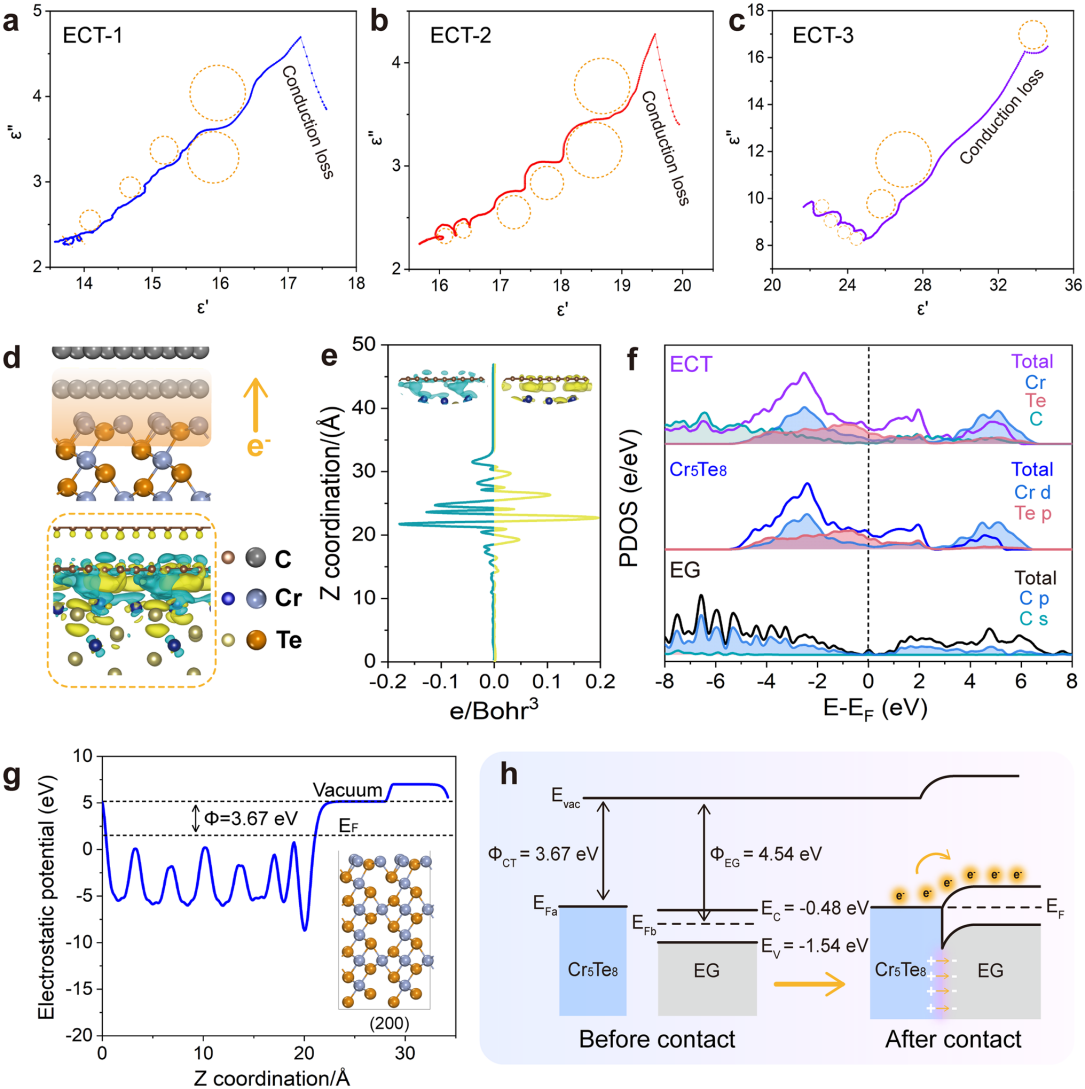
Interfacial polarization mechanism of ECTs. The Cole–Cole curves of ECT-1 (**a**), ECT-2 (**b**), and ECT-3 (**c**). DFT calculations of heterojunction (**d**–**h**). **d** The atomic model and the three-dimensional charge density difference of the heterostructure interface. The blue isosurface suggests a decrease of electron density, and the yellow isosurface depicts an increase of electron density (0.0003 electrons/Å). **e** *Z*-axis charge density difference of ECT in (**d**). **f** Projected density of state of EG, Cr_5_Te_8_, and ECT heterojunction, all the energies are referred to the Fermi level. **g** Work function of Cr_5_Te_8_ (200) surface. **h** Energy band alignment of Cr_5_Te_8_ and EG heterojunction before and after Mott–Schottky contact, vacuum energy (*E*_vac_), conduction band (*E*_c_), valence band (*E*_v_), Fermi level (*E*_F_) and work function (Φ), showing that the contact between Cr_5_Te_8_ and EG can trigger the redistribution of interfacial charge. (Color figure online)

- i. Heterointerfaces induced interfacial polarization. To investigate the polarization loss induced by interfacial interplay, Cole–Cole plots are provided in Fig. 4a-c. The relationship between the dissipation (*ε*″) and storage (ε′) capacity can be established with the help of the static permittivity, *ε*_s_, and permittivity at infinite high frequency, ε_∞_: (*ε*′ − *ε*_s_ + *ε*_∞_/2)^2^ + (*ε*′′)^2^ = ((*ε*_s_ − *ε*_∞_)/2)^2^ [45, 46]. Thus, the Debye semicircles in Cole–Cole curves signify the relaxation process. Apparently, compared to pure Cr_5_Te_8_ (Fig. S5a) and EG (Fig. S5b), ECTs show more Cole–Cole semicircles (Fig. 4a-c). This indicates that ECTs undergo the Debye relaxation process more prominently than pure Cr_5_Te_8_ and EG, which can be attributed to the interface polarization behaviors [47, 48]. Furthermore, the linear Cole–Cole curves are related to the conduction loss [49, 50].

To deeply understand the mechanisms of the interface enhanced the EMA capability of ECT heterojunctions, we conducted static DFT calculations to further investigate charge migration and distribution at the interface. Primarily, the (200) surface of Cr_5_Te_8_ crystals observed in Fig. 1 and the (001) surface of EG were selected for modeling, and the optimized structure is shown in Fig. 4d. Specifically, the contact between Cr_5_Te_8_ and EG forms a van der Waals heterojunction rather than interfacial atomic bonding. Moreover, electron density difference isosurfaces and corresponding integration along the z-axis of the heterojunction interface are displayed in Fig. 4d, e. Although the charge transfer at the interface is bidirectional with charge accumulation and depletion, Bader charge analysis shows that there is a net charge transfer of 0.6 |e| from Cr_5_Te_8_ to EG through the interface. Further, the projected density of states (PDOS) was inspected to analyze the charge transport of the ECT heterojunction (Fig. 4f). The energy bands of Cr_5_Te_8_ crystals and ECT heterojunction significantly cross the Fermi level, indicating their metallic nature. Besides, the band structure of the ECT heterojunction exhibits higher dispersion compared to that of Cr_5_Te_8_, leading to enhanced electronic conductivity and conduction loss [51]. It’s worth noting that carbon materials can exhibit properties of either a metal or a semiconductor under specific conditions [21, 52]. Therefore, we measured the band gap of EG using a UV–vis spectrophotometer, and the value is 1.06 eV (Fig. S6a). This result manifests that the absence of the EG bandgap in PDOS stems from the PBE function containing unphysical self-Coulomb repulsion, resulting in a systematic underestimate of the bandgap [53].

Intriguingly, the Mott–Schottky heterojunction is adapted to disclose the positive effect of the interface on the EMA capacity due to the heterostructure formed between metallic Cr_5_Te_8_ and semiconducting EG. The work function (Φ) of Cr_5_Te_8_ (200) and EG was calculated by UPS to be 3.67 and 4.54 eV, respectively, as shown in Fig. 4g and Fig. S6b-c. Figure 4h shows the interface coupling between Cr_5_Te_8_ and EG. Their band alignment generates a built-in electric field and Schottky barrier, benefitting intensive interface polarization loss.

**Fig. 5:**
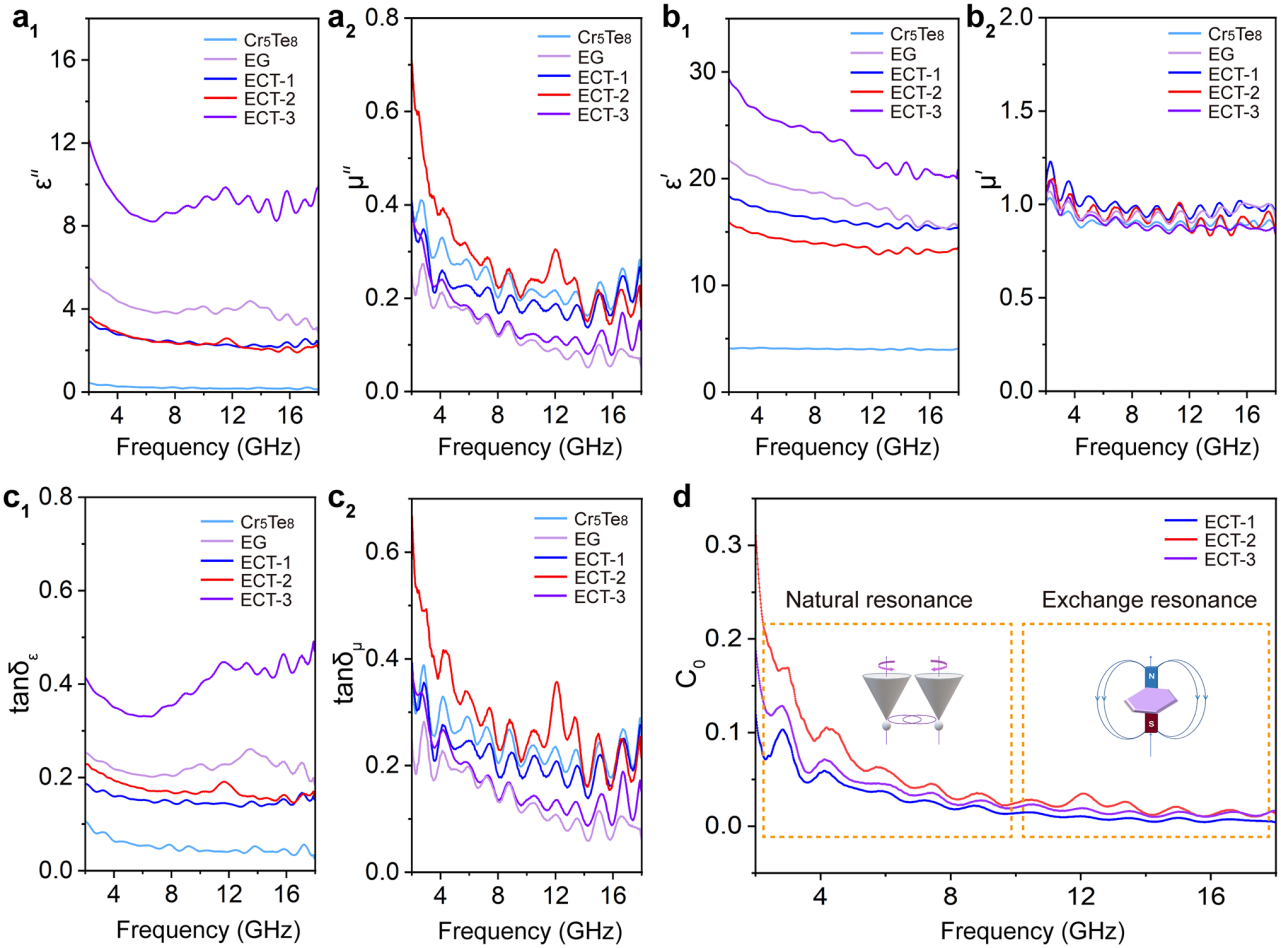
**a**_**1**_ Imaginary part and **b**_**1**_ real part of permittivity. **a**_**2**_ imaginary part and **b**_**2**_ real part of permeability. ECTs have an excellent impedance matching degree due to the appropriate electromagnetic parameters. **c**_**1**_ dielectric and **c**_**2**_ magnetic loss tangents. ECTs possess excellent dielectric and magnetic loss capabilities. **d** Coefficient (*C*_0_) values of ECTs. The magnetic loss of ECTs primarily generates from natural resonance and exchange resonance. (Color figure online)

- ii. Magnetic-dielectric synergistic effect. This combined effect of dielectric loss and magnetic loss is mainly evaluated by electromagnetic parameters and their derived loss parameters. The complex permittivity (*ε*′ and *ε*″) and the complex permeability (*µ*′ and *µ*″) with frequency-dependent fluctuations are depicted in Fig. 5a, b. The ε′ values of ECT and EG exhibit a decreasing trend as the frequency increases, which is related to the frequency dispersion behavior caused by polarization hysteresis under a high-frequency electric field [30]. The *ε*″ values are associated with the dissipation potential of electric field energy [54]. Compared with Cr_5_Te_8_, both ECT and EG display higher ε″ values, indicating the introduction of porous expanded graphite can enhance electron migration efficiency and further enhance electromagnetic energy dissipation. The inconspicuous variation of µ′ indicates the relaxation of magnetic moments precession rather than magnetic hysteresis, while the fluctuated µ″ values show the strong magnetic dissipation capability. Additionally, as shown in Fig. 5c1, the calculated dielectric loss tangent (tan*δ*_*ε*_ = *ε*″/*ε*′, where the tan*δ*_*ε*_ value is positively correlated with the dielectric loss capacity) values of all samples exhibit the same trends as *ε*″ values, confirming the stronger attenuation capability of ECTs to electric field energy [29, 55]. Similarly, the superior magnetic loss capacity of ECTs and Cr_5_Te_8_ is evident in the higher magnetic loss tangent (tan*δ*_*μ*_ = *μ*″/*μ*′) values in Fig. 5c2 [55], primarily due to the robust magnetic coupling interaction stemming from the spin polarization of Cr_5_Te_8_ nanoflakes [17]. When microwaves propagate into the Cr_5_Te_8_/graphite composite, the high-frequency magnetic field component induces spin precession in the Cr_5_Te_8_ nanoflakes [56]. Their stray magnetic fields can affect neighboring Cr_5_Te_8_ through corresponding dynamic variation [57]. As a result, a mutual induction process occurs between adjacent Cr_5_Te_8_ nanoflakes, achieving the interparticle coupling interaction. Therefore, these magnetic coupling interactions among Cr_5_Te_8_ nanoflakes effectively interact with microwaves to achieve electromagnetic microwave attenuation. Furthermore, magnetic loss depends significantly on eddy current loss, natural resonance, and exchange resonance. As shown in the coefficient curves (*C*_0_ = *μ*"(*μ*')^−2^*f*^−1^) [58, 59], the C_0_ values of ECTs exhibit significant fluctuations in 2–10 GHz, indicating natural resonance occurring at the low frequency (Fig. 5d) [19, 59]. The several fluctuations located at high-frequency regions (especially > 10 GHz) are attributed to exchange resonance [7, 60, 61]. Briefly, the introduction of the Cr_5_Te_8_ component can effectively induce exchange resonance loss and natural resonance loss, which are the main sources of magnetic loss resulting from magnetic coupling interaction, pinning effect, and spin wave excitation. Therefore, it can be concluded that the magnetoelectric synergistic loss mechanism of ECTs with a heterostructure plays a vital role in improving microwave absorption capacity.
- iii. Multiple reflection and interlayer scattering. The vertical spatial distribution of ECT allows multiple reflections and scatterings to trap electromagnetic microwaves. Moreover, the porous EG microstructure is verified by the N_2_ adsorption–desorption isotherm curves (Fig. S7). The pore structure facilitates the reduction of absorber density and the appropriate specific surface area is conducive to improving the impedance matching and interfacial polarization, thereby promoting microwave attenuation [22].
- iv. Conduction loss caused by conductive networks**.** The randomly distributed Cr_5_Te_8_ units contribute to the construction of a spatial conductive network and an increase of conductive pathways [1, 62]. When electromagnetic microwaves permeate the network, the excited electrons migrate along the axial direction or to other neighboring lamellas [22]. This process leads to the transformation of electromagnetic energy into thermal energy due to the resistance of the absorber.

**Fig. 6:**
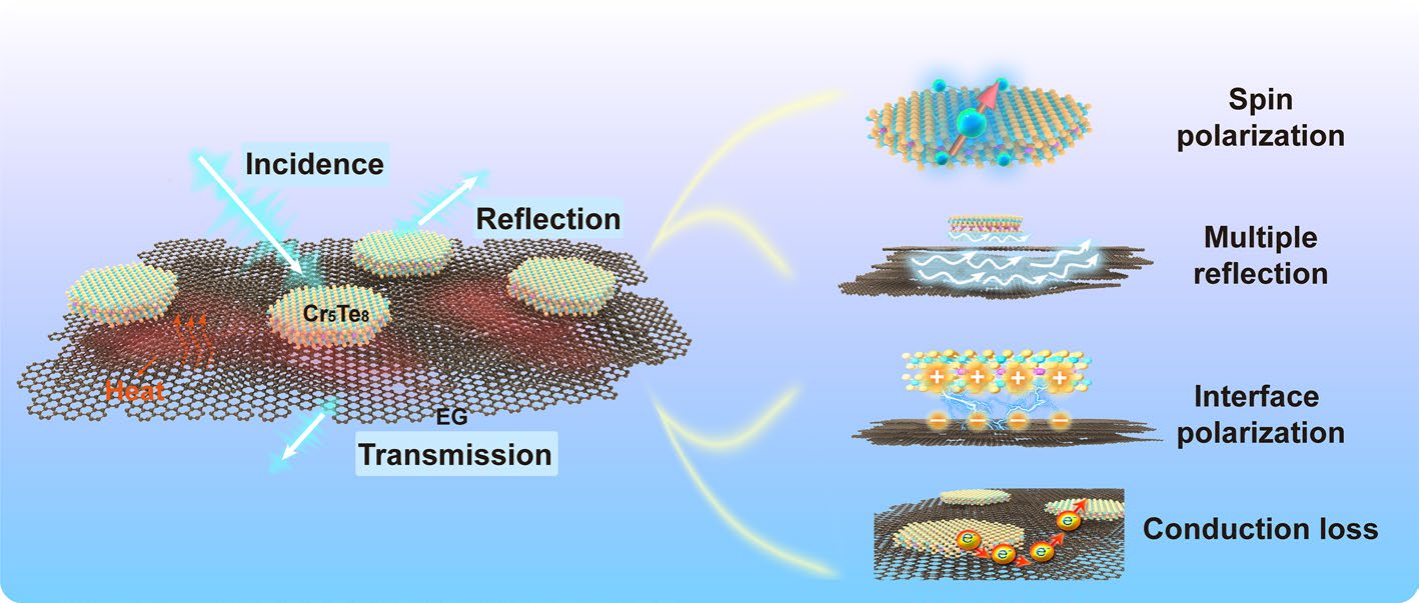
Multivariate synergistic loss mechanism of the ECT absorber. Polarization, conduction, and magnetic-dielectric synergy losses are mainly included. (Color figure online)

Figure 6 generalizes the multivariate synergistic loss mechanism of the ECT absorber, which includes the generous interface polarization loss brought about by the heterostructure. Subsequently, the abundant micro-conducting network brings the conductive loss. Additionally, the structural advantages, including the ample heterointerface, the layered structure of Cr_5_Te_8_, and the porous EG, lead to multi-level reflection and interlayer scattering of electromagnetic microwaves. Meanwhile, the strong atom spin effect of Cr_5_Te_8_ enhances the magnetic loss and optimizes impedance matching of ECT. These synergistic effects result in the powerful EMA.

### Excellent RCS Reduction Ability

**Fig. 7:**
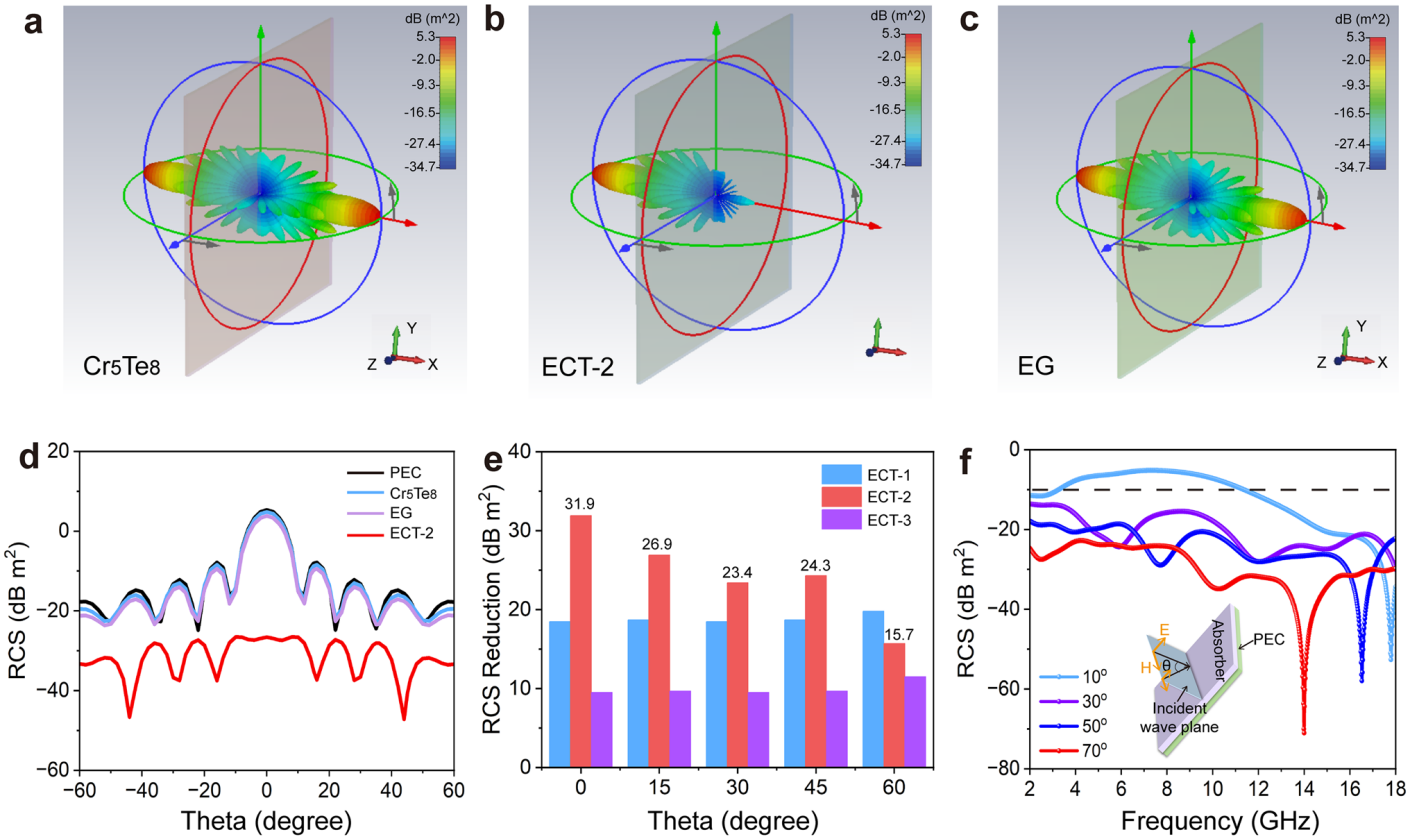
RCS simulations. The three-dimensional radar microwave scattering signals of **a** Cr_5_Te_8_, **b** ECT-2, and **c** EG, respectively, showing that ECT-2 has a better radar signal scattering ability. **d** RCS simulated curves at different incident thetas of PEC and Cr_5_Te_8_, EG and ECT-2. **e** The RCS reduction values of ECTs. **f** Frequency dependences of ECT-2 RCS values at different incident thetas. Inset is the schematic diagram of CST simulation, showing the excellent radar signal scattering ability of ECT-2 under different incident thetas. (Color figure online)

With the rapid development of stealth technology, more attentions have been focused on the RCS which can evaluate the anti-detection ability of the target. In general, the lower RCS corresponds to the stronger anti-detection ability [63]. Notably, EMA materials can effectively reduce RCS due to the efficient attenuation of incident electromagnetic waves [64]. Therefore, to assess the practical electromagnetic loss potential of ECTs under near-practical situations, the RCS of the as-prepared ECTs in the far-field response range was simulated using CST software, as shown in Fig. 7. This simulation provides insights into the performance of absorbing materials under more realistic conditions. The 3D radar scattering results in Fig. 7a-c reveal that EG and Cr_5_Te_8_ present the largest radar scattering signals among those tested, which is detrimental for stealth applications. In sharp contrast, ECT-2 exhibits the lowest radar scattering signals, indicating superior stealth performance. To further evaluate their performance, the simulated RCS values within a scanning angle range of –60° to 60° at 15.4 GHz (Ku band) of PEC and ECT-covered PEC are shown in Figs. 7d and S8. Obviously, the RCS peak values decrease when θ is changed from 0° to ± 60°, proving scanning angles affect the displayed RCS. Besides, the RCS values of PEC, EG, and Cr_5_Te_8_ are basically coincidental and are larger than other composite coatings, indicating that ECT-1, ECT-2, and ECT-3 coatings have a great contribution to electromagnetic microwave attenuations. In addition, when the incident angle of the electromagnetic microwave is greater than 20°, the RCS value of all coatings is less than − 10 dB, suggesting that radar reflections with large incident angles account for a smaller proportion. Therefore, it is important to develop materials that have good absorption even at small incident angles. To separately analyze the specific contribution of ECTs to RCS values, the RCS reduction values in small angles can be obtained in Fig. 7e by calculating the RCS values of ECTs, except PEC. Figure 7e reveals that the RCS contribution of ECTs is basically greater than 10 dB  m^2^. When the incident angle is 0°, the RCS contribution of ECT-2 can reach 31.9 dB m^2^, which is superior than previous reports [5, 18, 65]. For electromagnetic microwaves incident at 0–60°, the RCS values of ECT-1 and ECT-2 all exceed 15 dB m^2^, demonstrating the excellent EMA characteristics of ECT. Moreover, the corresponding RCS value of the representative incident angle of ECT-2 at 1.4 mm is also statistically analyzed (Fig. 7f). The results show that the RCS value remains consistently below − 10 dB m^2^ when the incident angle exceeds 30°, highlighting the material coating has great electromagnetic attenuation characteristics. Undoubtedly, ECT-absorbing coatings can effectively reduce RCS and play an irreplaceable role in the system of protecting high-value targets.

## Conclusions

In summary, we have successfully constructed ECTs by assembling Cr_5_Te_8_ nanoflakes and porous dielectric EG via CVD. As a result, an optimal RL of − 57.6 dB is achieved in a 1.4 mm film with a low filling rate of 10%, which is mainly attributed to the dielectric polarization induced by the charge redistribution at the interfaces. Meanwhile, the RCS reduction of ECT coating can reach 31.9 dB m^2^, and the RCS value is almost less than − 10 dB m^2^ when the incident angle is greater than 30°, demonstrating great electromagnetic microwave scattering ability and radar stealth capability. The powerful combination of magnetic-dielectric integrated compositions, strong interface polarization relaxation induced by heterogeneous interfaces, and abundant interlayer scattering, lead to the promoted impedance matching and superior EMA properties of ECTs. This work provides new perspectives for designing 2D TMC-based EMA materials to achieve essential breakthroughs in mechanistic investigations in this promising field.

## Supplementary Information

Below is the link to the electronic supplementary material.

Supplementary file1 (PDF 977 KB)
