# Supplementary material for: Two-Dimensional Cr5Te8@Graphite Heterostructure for Efficient Electromagnetic Microwave Absorption
Source: Nanomicro Lett. 2023 Dec 20;16:60. doi: 10.1007/s40820-023-01271-7 (PMC10733264; doi:10.1007/s40820-023-01271-7)
Supplement: Supplementary file 1 — Supplementary file1 (PDF 977 KB) [file 40820_2023_1271_MOESM1_ESM.pdf]

Supporting Information for

Two-Dimensional Cr_5Te_8@Graphite Heterostructure for Efficient Electromagnetic Microwave Absorption

Liyuan Qin[1, 2], Ziyang Guo[1], Shuai Zhao[2, *], Denan Kong[2], Wei Jiang[2], Ruibin Liu[2], Xijuan Lv[2, *], Jiadong Zhou[2, 3, *], and Qinghai Shu[1, 4, *]

[1] School of Materials Science and Engineering, Beijing Institute of Technology, Beijing 100081, P. R. China

[2] Centre for Quantum Physics, Key Laboratory of Advanced Optoelectronic Quantum Architecture and Measurement (MOE), School of Physics, Beijing Institute of Technology, Beijing 100081, P. R. China

[3] Advanced Research Institute of Multidisciplinary Science, Beijing Institute of Technology, Beijing 100081, P. R. China

[4] Tangshan Research Institute, Beijing Institute of Technology, Tangshan 063099, P. R. China

*Corresponding authors. E-mail: zhaoshuai117@bit.edu.cn (Shuai Zhao); lvxj@bit.edu.cn (Xijuan Lv); jdzhou@bit.edu.cn (Jiadong Zhou); qhshu121@bit.edu.cn (Qinghai Shu)

Supplementary Figures and Tables

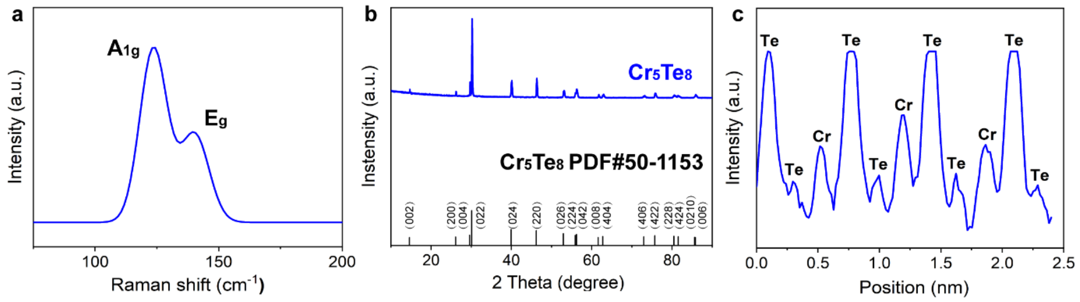

Fig. S1 Structure of Cr_5Te_8 crystals. **a** Raman spectra and **b** XRD pattern of Cr_5Te_8. **c** Intensity line profile of the rectangle marked in **Fig. 1c**, showing the high quality of Cr_5Te_8 crystals

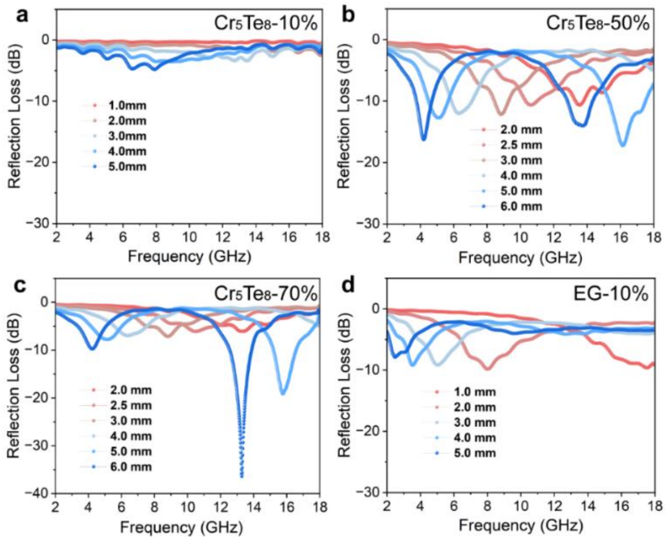

Fig. S2 Reflection loss curves of Cr_5Te_8 and EG at different thicknesses and different loadings. **a–c** RL values at 10 wt% loadings (**a**), 50 wt% loadings (**b**), and 70 wt% (**c**) loadings of Cr_5Te_8, showing the dependence of EMA performance on the high loading rate. **d** RL values at 10 wt% loadings of EG, showing the poor EMA performance of EG at low loading

Table S1 Comparison of the minimum RL (RL_{min}) and corresponding thickness of different materials

Materials	RL_{min} (dB)	Thickness (mm)	Refs.
High-entropy CrO_X	−30.7	3.2	[S1]
SiC/SiO_2	−54.68	4.49	[S2]
Fe/FeO_x@C	−28.9	2.0	[S3]
$NiSe_2$/FeSe@NC	−52.8	2.1	[S4]
$Ti_3C_2T_x/MoS_2$	−52.1	4.1	[S5]
CoNi/MnO@C	−55.2	2.6	[S6]
$NiFe_2O_4$-G	−48.1	3.0	[S7]
CoFe@NC/rGO	−53.0	2.4	[S8]
$MoSe_2$@rGO	−56.9	5.67	[S9]
PC/Fe_3O_4@PDA	−46.67	2.4	[S10]
rGO/Cu/Fe_3O_4	−18.3	3.1	[S11]
C@MoO_2/G	−33.5	5.0	[S12]
CoFe-CNT-rGO	−56.1	2.73	[S13]
Cu/C@MoS_2	−48.22	2.5	[S14]
FeCo/C	−35.9	4.0	[S15]
**Cr_5Te_8@EG**	**−57.6**	**1.4**	**This work**

Table S2 Comparison of the RL_{min} and corresponding loading of different materials

Materials	RL_{min} (dB)	Loading (wt %)	Refs.
$Fe/FeO_x@C$	−28.9	60	[S3]
$rGO/Cu/Fe_3O_4$	−18.3	55	[S11]
Ni@C-CoNi	−51.4	50	[S16]
BCN/C/Co	−56.7	48	[S17]
$MoSe_2@rGO$	−56.9	40	[S9]
CoFe@NC/rGO	−53.0	35	[S8]
$C@MoO_2/G$	−33.5	30	[S12]
$PC/Fe_3O_4@PDA$	−46.67	30	[S10]
$Cu/C@MoS_2$	−48.22	30	[S14]
FeCo/C	−35.9	30	[S15]
$NiO/NiFe_2O_4@NG$	−52.61	25	[S18]
$NiFe_2O_4$-G	−48.1	20	[S7]
$WS_2/CoS_2@CCF$	−51.26	20	[S19]
$CoO/FeCo_2O_4/Ti_3C_2T_x$	−41.06	10	[S20]
**$Cr_5Te_8@EG$**	**−57.6**	**10**	**This work**

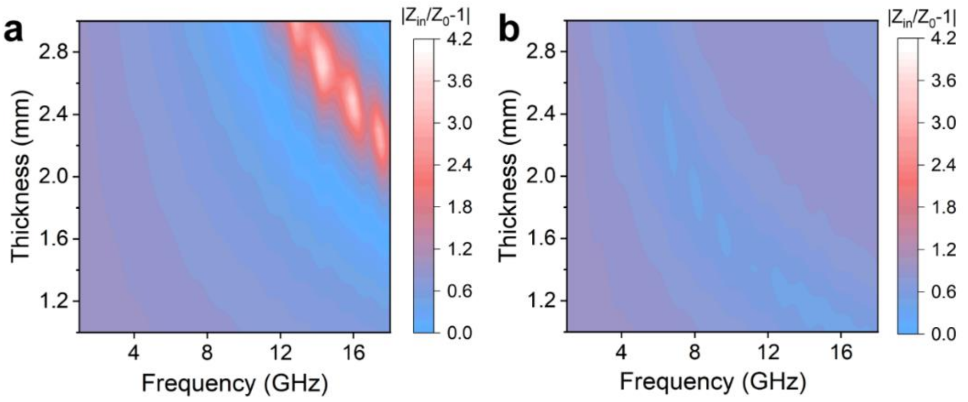

Fig. S3 Impedance matching capability of Cr_5Te_8 and EG. Impedance matching curves under different thicknesses of Cr_5Te_8 **a**, EG **b**. The Z values of Cr_5Te_8 are far larger than the value of 1 and the Z values of EG are far small than the value of 1, indicating their poor impedance matching capability

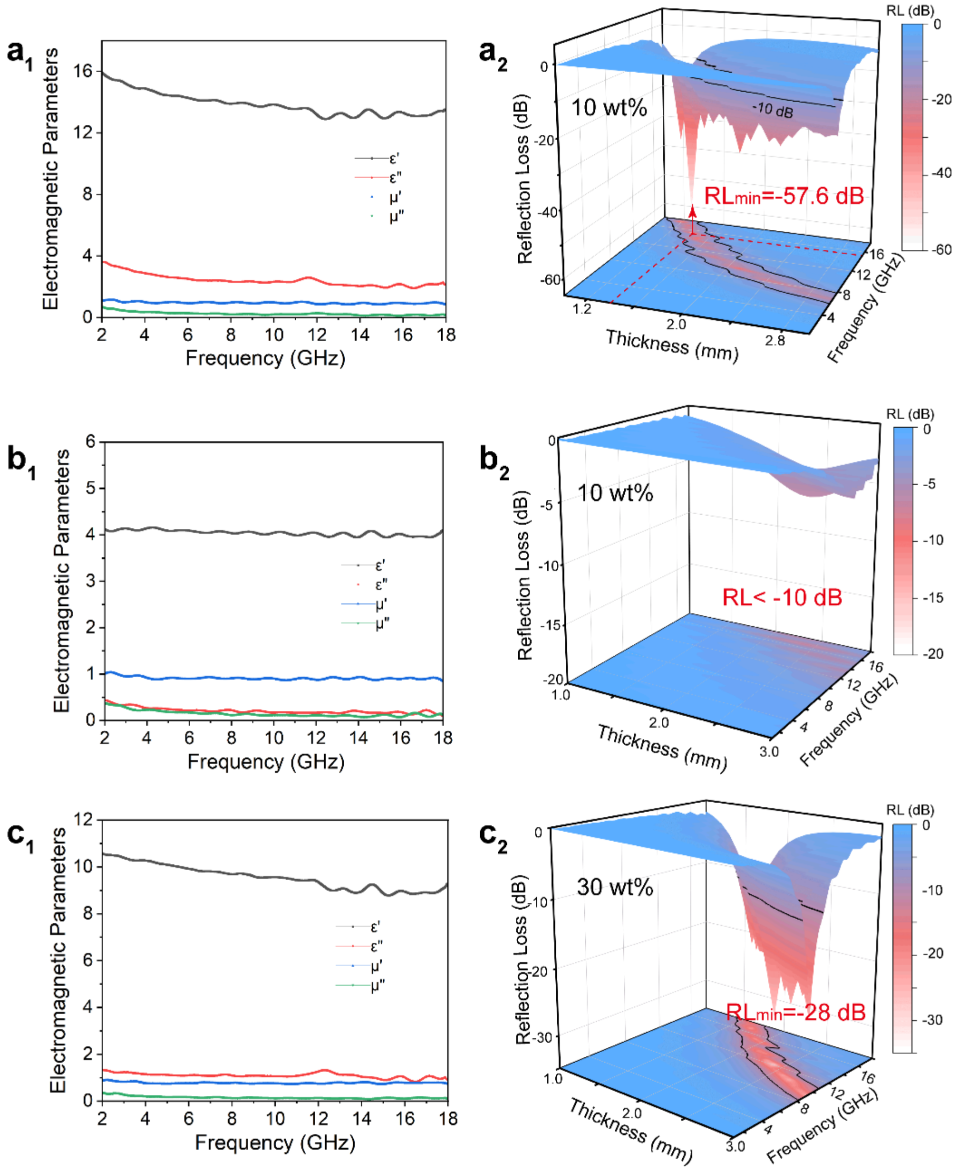

Fig. S4 a$_1$ the electromagnetic parameters, and **a**$_2$ reflection loss of Cr_5Te_8@expanded graphite heterojunction under 10% of loading rate. **b**$_1$ the electromagnetic parameters, and **b**$_2$ reflection loss of Cr_5Te_8 physically mixed with expanded graphite under 10% of loading rate. **c**$_1$ the electromagnetic parameters, and **c**$_2$ reflection loss of Cr_5Te_8 physically mixed with expanded graphite under 30% of loading rate. Note: Cr_5Te_8@expanded graphite heterojunction is synthesized using a 1:1 mass ratio of raw materials ($CrCl_3$ to unexpanded graphite). Cr_5Te_8 physically mixed with expanded graphite is prepared using 1:1 mass ratio of Cr_5Te_8 to expanded graphite

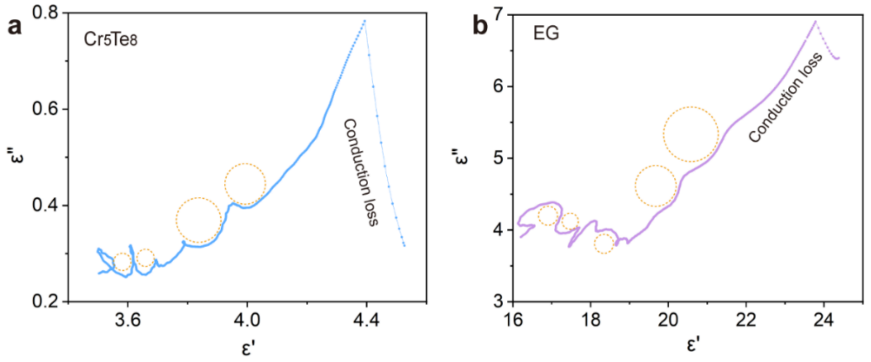

Fig. S5 Polarization relaxation ability of Cr_5Te_8 and EG. Cole-Cole curves of Cr_5Te_8 **a** and EG **b**, respectively. More Cole-Cole semicircles appear in ECTs than those for Cr_5Te_8 and EG, showing the significance of hetero-interface polarization behaviors of ECTs

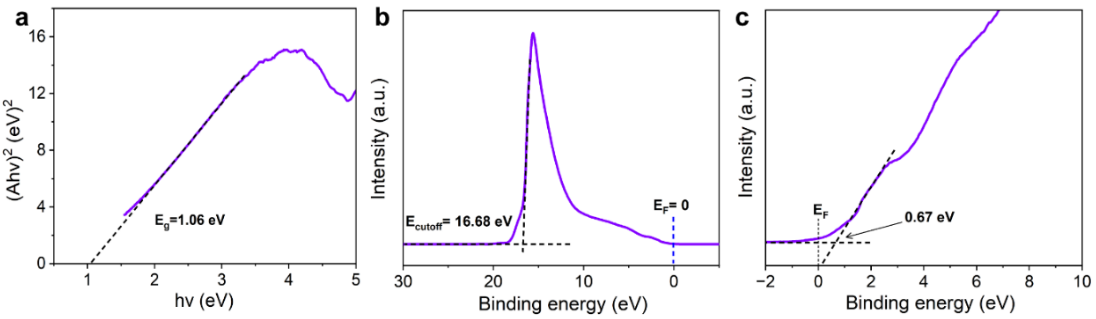

Fig. S6 Characterization of EG band structure. The **a** bandgap, **b** work function, and **c** valence band top of EG

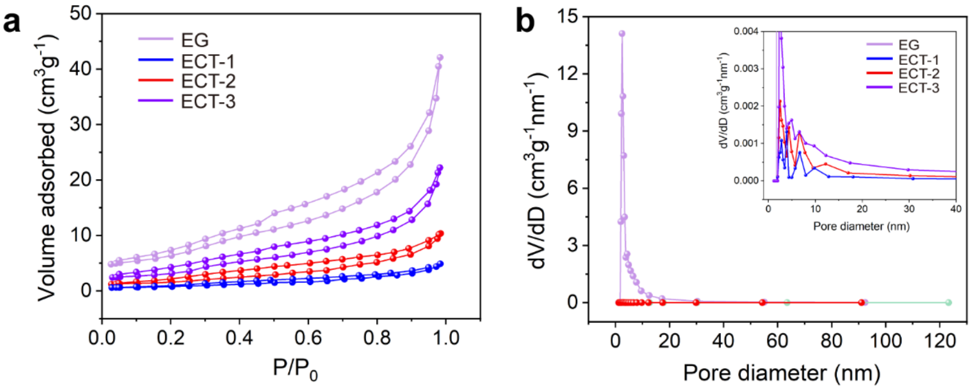

Fig. S7 Specific surface area and pore structure of ECT-1, ECT-2, ECT-3, and EG. **a** N_2 adsorption-desorption isotherm curves of ECT and EG, showing the influence of light and porous EG content on the specific surface area. **b** Pore size distribution curves of ECT and EG

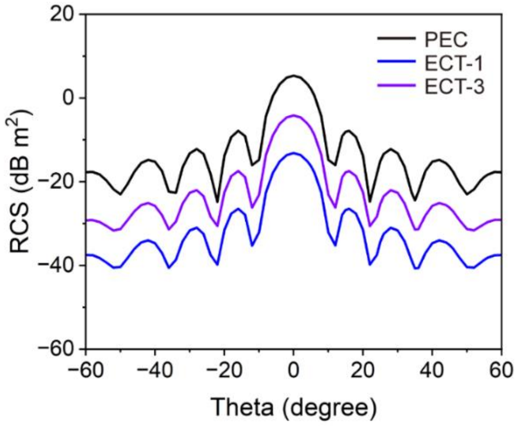

Fig. S8 RCS simulation of ECT-1 and ECT-3. RCS simulated curves at different incident thetas of PEC, ECT-1, and ECT-3, displaying that ECT-1 has a superior contribution to EMA attenuations than ECT-3

Supplementary References

[S1] B. Zhao, Y. Du, Z. Yan, L. Rao, G. Chen et al., Structural defects in phase-regulated high-entropy oxides toward superior microwave absorption properties. Adv. Funct. Mater. **33**(1), 2209924 (2023). https://doi.org/10.1002/adfm.202209924

[S2] Z. Xiang, Y. Wang, X. Yin, Q. He, Microwave absorption performance of porous heterogeneous SiC/SiO_2 microspheres. Chem. Eng. J. **451**(part 2), 138742 (2023). https://doi.org/10.1016/j.cej.2022.138742

[S3] M. Zhang, L. Wang, S. Bao, Z. Song, W. Chen et al., A finite oxidation strategy for customizing heterogeneous interfaces to enhance magnetic loss ability and microwave absorption of Fe-cored carbon microcapsules. Nano Res. 16, 11084–11095 (2023). https://doi.org/10.1007/s12274-023-5511-7

[S4] Z. Yang, T. Wang, J. Wang, Z. Luo, Q. Zhang et al., Heterogeneous N-doped carbon composite $NiSe_2$-FeSe double-shell hollow nanorods for tunable and high-efficient microwave attenuation. Carbon **201**, 491–503 (2023). https://doi.org/10.1016/j.carbon.2022.09.023

[S5] M. Li, W. Zhu, X. Li, H. Xu, X. Fan et al., $Ti_3C_2T_x$/MoS_2 self-rolling rod-based foam boosts interfacial polarization for electromagnetic wave absorption. Adv. Sci. **9**(16), 2201118 (2022). https://doi.org/10.1002/advs.202201118

[S6] Y. Liu, X. Zhou, Z. Jia, H. Wu, G. Wu, Oxygen vacancy-induced dielectric polarization prevails in the electromagnetic wave-absorbing mechanism for Mn-based MOFs-derived composites. Adv. Funct. Mater. **32**(34), 2204499 (2022). https://doi.org/10.1002/adfm.202204499

[S7] Y. Wang, L. Yao, Q. Zheng, M.-S. Cao, Graphene-wrapped multiloculated nickel ferrite: A highly efficient electromagnetic attenuation material for microwave absorbing and green shielding. Nano Res. **15**(7), 6751–6760 (2022). https://doi.org/10.1007/s12274-022-4428-x

[S8] S. Wei, T. Chen, Z. Shi, S. Chen, Preparation of CoFe@N-doped C/rGO composites derived from cofe prussian blue analogues for efficient microwave absorption. J. Colloid Interf. Sci. **610**, 395–406 (2022). https://doi.org/10.1016/j.jcis.2021.12.051

[S9] X. Fu, Q. Zheng, L. Li, M. Cao, Vertically implanting $MoSe_2$ nanosheets on the rGO sheets towards excellent multi-band microwave absorption. Carbon **197**, 324–333 (2022). https://doi.org/10.1016/j.carbon.2022.06.037

[S10] Y. Xie, Y. Guo, T. Cheng, L. Zhao, T. Wang et al., Efficient electromagnetic wave absorption performances dominated by exchanged resonance of lightweight PC/Fe_3O_4@PDA hybrid nanocomposite. Chem. Eng. J. **457**, 141205 (2023). https://doi.org/10.1016/j.cej.2022.141205

[S11] G. Fang, C. Liu, Y. Yang, K. Peng, Y. Cao et al., Regulating percolation threshold via dual conductive phases for high-efficiency microwave absorption performance in C and X bands. ACS Appl. Mater. Interfaces **13**(31), 37517–37526 (2021). https://doi.org/10.1021/acsami.1c10110

[S12] C. Wu, Z. Chen, M. Wang, X. Cao, Y. Zhang et al., Confining tiny MoO_2 clusters into reduced graphene oxide for highly efficient low frequency microwave absorption. Small **16**(30), 2001686 (2020). https://doi.org/10.1002/smll.202001686

[S13] R. Meng, T. Zhang, X. Liu, P. Jiao, J. Li et al., Graphene oxide-assisted Co-sintering synthesis of carbon nanotubes with enhanced electromagnetic wave absorption performance. Carbon **185**, 186–197 (2021). https://doi.org/10.1016/j.carbon.2021.09.018

[S14] Z. Gao, Z. Ma, D. Lan, Z. Zhao, L. Zhang et al., Synergisticpolarization loss of MoS_2-based multiphase solid solution for electromagnetic wave absorption. Adv. Funct. Mater. **32**(18), 2112294 (2022). https://doi.org/10.1002/adfm.202112294

[S15] L. Rao, L. Wang, C. Yang, R. Zhang, J. Zhang et al., Confined diffusion strategy for customizing magnetic coupling spaces to enhance low-frequency electromagnetic wave absorption. Adv. Funct. Mater. **33**(16), 2213258 (2023). https://doi.org/10.1002/adfm.202213258

[S16] M. Sun, D. Wang, Z. Xiong, Z. Zhang, L. Qin et al., Multi-dimensional Ni@C-CoNi composites with strong magnetic interaction toward superior microwave absorption. J. Mater. Sci. Technol. **130**, 176–183 (2022). https://doi.org/10.1016/j.jmst.2022.05.016

[S17] H. Tian, J. Qiao, Y. Yang, D. Xu, X. Meng et al., ZIF-67-derived Co/C embedded boron carbonitride nanotubes for efficient electromagnetic wave absorption. Chem. Eng. J. **450**(part 1), 138011 (2022). https://doi.org/10.1016/j.cej.2022.138011

[S18] J. Luo, H. Guo, J. Zhou, F. Guo, G. Liu et al., Rational construction of heterogeneous interfaces for bimetallic MOFs-derived/rGO composites towards optimizing the electromagnetic wave absorption. Chem. Eng. J. **429**, 132238 (2022). https://doi.org/10.1016/j.cej.2021.132238

[S19] Y. Guo, H. Liu, D. Wang, Z.M. El-Bahy, J.T. Althakafy et al., Engineering hierarchical heterostructure material based on metal-organic frameworks and cotton fiber for high-efficient microwave absorber. Nano Res. **15**(8), 6841–6850 (2022). https://doi.org/10.1007/s12274-022-4533-x

[S20] H. Wang, X. Sun, Y. Xin, S. Yang, P. Hu et al., Ultrathin self-assembly MXene/Co-based bimetallic oxide heterostructures as superior and modulated microwave absorber. J. Mater. Sci. Technol. **134**, 132–141 (2023). https://doi.org/10.1016/j.jmst.2022.05.061
